# (2*E*)-3-(Dimethyl­amino)-1-(4-fluoro­phen­yl)prop-2-en-1-one

**DOI:** 10.1107/S160053681204202X

**Published:** 2012-10-13

**Authors:** Rajni Kant, Vivek K. Gupta, Kamini Kapoor, Madhukar B. Deshmukh, D. R. Patil, P. V. Anbhule

**Affiliations:** a X-ray Crystallography Laboratory, Post-Graduate Department of Physics & Electronics, University of Jammu, Jammu Tawi 180 006, India; b Department of Chemistry, Shivaji University, Kolhapur 416 004, India

## Abstract

In the title compound, C_11_H_12_FNO, the dihedral angle between the prop-2-en-1-one group and the benzene ring is 19.33 (6)°. The configuration of the keto group with respect to the olefinic double bond is *s-cis*. In the crystal, the mol­ecules form dimers through aromatic π–π stacking inter­actions [centroid–centroid distance = 3.667 (1) Å] and are linked *via* C—H⋯O inter­actions into chains along the *b* axis.

## Related literature

For the synthesis and pharmaceutical activity of enamino­nes, see: Kantevari *et al.* (2007 ▶); Ke *et al.* (2009 ▶); Omran *et al.* (1997 ▶); Eddington *et al.* (2003 ▶). For a related structure, see: Deng *et al.* (2010 ▶).
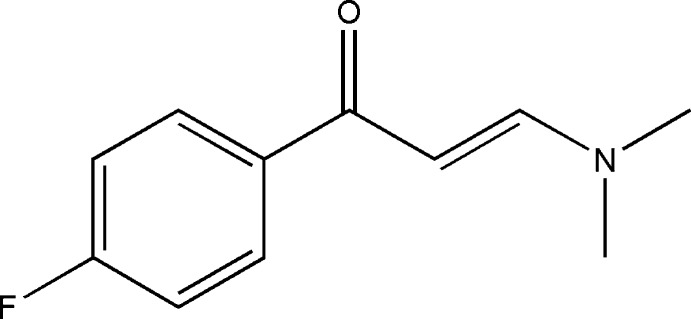


## Experimental

### 

#### Crystal data

C_11_H_12_FNO
*M* *_r_* = 193.22
Monoclinic, 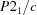

*a* = 13.2832 (6) Å
*b* = 5.8530 (2) Å
*c* = 14.2995 (8) Å
β = 116.086 (6)°
*V* = 998.49 (8) Å^3^
*Z* = 4
Mo *K*α radiation
μ = 0.10 mm^−1^
*T* = 293 K
0.3 × 0.2 × 0.2 mm

#### Data collection

Oxford Diffraction Xcalibur Sapphire3 diffractometer
Absorption correction: multi-scan (*CrysAlis PRO*; Oxford Diffraction, 2010 ▶) *T*_min_ = 0.824, *T*_max_ = 1.000
14183 measured reflections
1952 independent reflections
1430 reflections with *I* > 2σ(*I*)
*R*_int_ = 0.040

#### Refinement

*R*[*F* ^2^ > 2σ(*F* ^2^)] = 0.046
*wR*(*F* ^2^) = 0.133
*S* = 1.04
1952 reflections
129 parameters
H-atom parameters constrained
Δρ_max_ = 0.20 e Å^−3^
Δρ_min_ = −0.16 e Å^−3^

### 

Data collection: *CrysAlis PRO* (Oxford Diffraction, 2010 ▶); cell refinement: *CrysAlis PRO*; data reduction: *CrysAlis PRO*; program(s) used to solve structure: *SHELXS97* (Sheldrick, 2008 ▶); program(s) used to refine structure: *SHELXL97* (Sheldrick, 2008 ▶); molecular graphics: *ORTEP-3* (Farrugia, 1997 ▶); software used to prepare material for publication: *PLATON* (Spek, 2009 ▶).

## Supplementary Material

Crystal structure: contains datablock(s) I, New_Global_Publ_Block. DOI: 10.1107/S160053681204202X/gk2520sup1.cif

Structure factors: contains datablock(s) I. DOI: 10.1107/S160053681204202X/gk2520Isup2.hkl

Supplementary material file. DOI: 10.1107/S160053681204202X/gk2520Isup3.cml

Additional supplementary materials:  crystallographic information; 3D view; checkCIF report

## Figures and Tables

**Table 1:** Hydrogen-bond geometry (Å, °)

*D*—H⋯*A*	*D*—H	H⋯*A*	*D*⋯*A*	*D*—H⋯*A*
C6—H6*B*⋯O1^i^	0.96	2.59	3.531 (3)	168

Symmetry code: (i) 
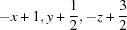
.

**Fig. 1.**
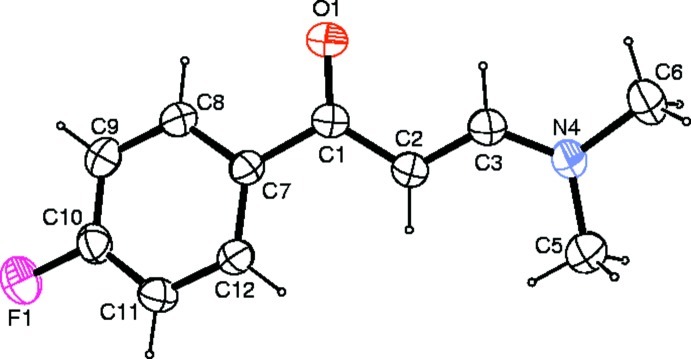
ORTEP view of the molecule with the atom-labeling scheme. The displacement ellipsoids are drawn at the 40% probability level. H atoms are shown as small spheres of arbitrary radii.

**Fig. 2.**
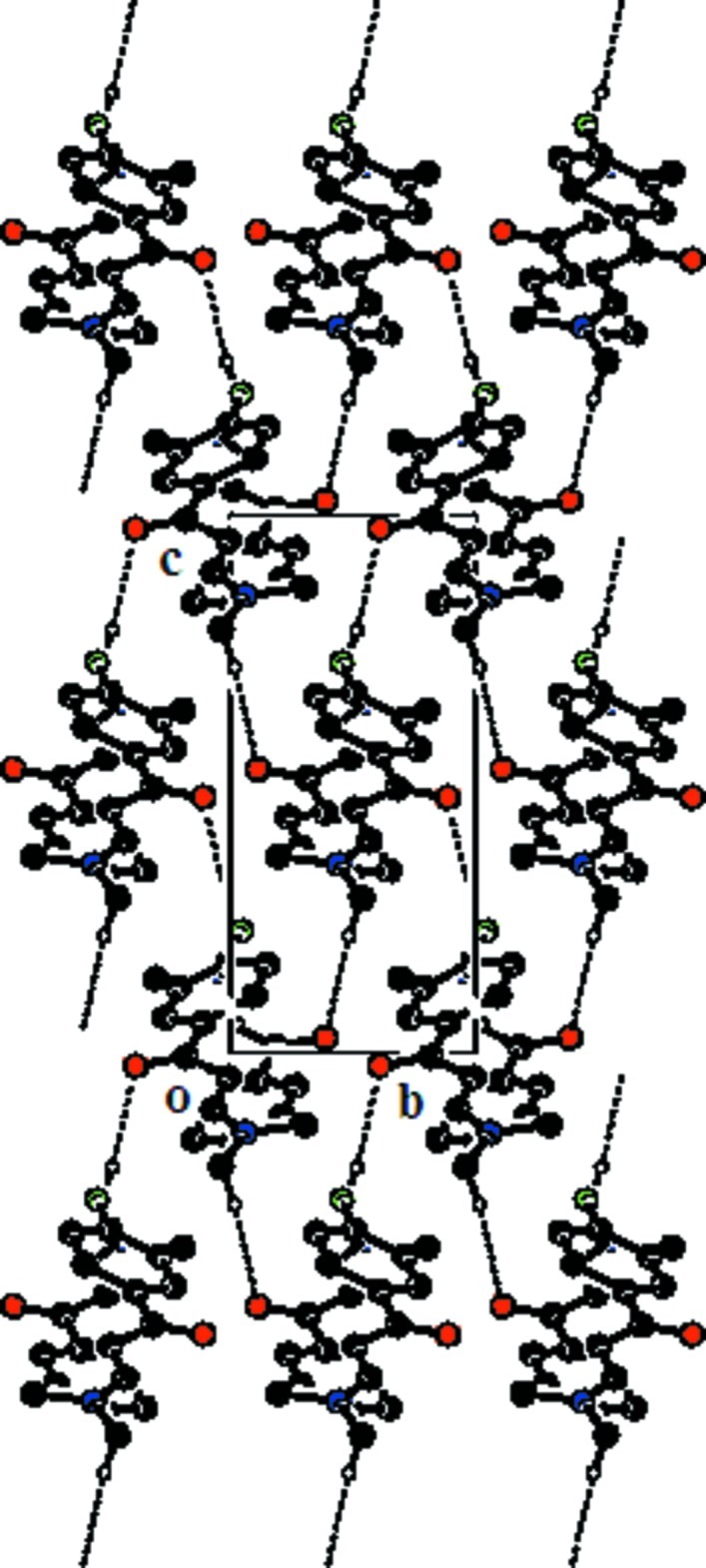
The packing arrangement of molecules viewed down the a axis. The broken lines show the intermolecular C—H···O interactions.

## supplementary crystallographic information

### Comment

(2E)-3-(Dimethylamino)-1-(4-fluorophenyl)prop-2-en-1-one is a versatile substrate used for synthesis of number of heterocyclic compounds and drug intermediates (Kantevari *et al.*, 2007; Ke *et al.*, 2009; Omran *et al.*, 1997; Eddington *et al.*, 2003).

The molecular structure of the title compound (I) is shown in Fig.1. The bond lengths and angles observed in (I) show normal values and are comparable with a related structure (Deng *et al.*, 2010). The dihedral angle between prop-2-en-1-one group and the phenyl ring is 19.33 (6) °. Molecules in the unit cell are packed together to form one dimensional assembly along the *b* axis (Fig.2) through intermolecular C6—H6B···O1 interactions (Table 1). The crystal structure is further stabilized by π–π interactions between the benzene ring (C7—C12) of the molecule at (*x*, *y*, *z*) and the benzene ring of an inversion related molecule at (- *x*, - *y*, -*z*)[centroid separation = 3.667 (1) Å, interplanar spacing = 3.535 Å and centroid shift = 0.97 Å].

### Experimental

In a 50 ml round bottom flask charged with 5 mmole of 4-methyl acetophenone and 5 mmole of dimethylformamide dimethyl acetal. Then 10 ml toulene was added and the reaction mixture was stirred for 3 h at 110°C. The reaction was monitored by TLC. After completion of reaction and cooling the reaction mixture was evaporated under vacuum. Finally, the product was isolated by column chromatography using ethyl acetate and n-hexane(2/8 vol.). Yield: 85%. IR(KBr): 1643, 1598, 1550,1439 cm-1. ^1^H NMR (300 MHz, CDCl3): 2.93(s, 3H, N—CH3), 3.15(s, 3H, N—CH3), 5.65–5.69 (d, 1H, =CH), 7.79–7.83(d, 1H,=CH), 7.05–7.11(m, 2*H*, Ar—H), 7.89–7.94 (m, 2H, Ar—H).

### Refinement

All H atoms were positioned geometrically and were treated as riding on their parent C atoms, with C—H distances of 0.93–0.96 Å and with *U*_iso_(H) = 1.2*U*_eq_(C) or 1.5*U*_eq_(methyl C).

### Crystal data

**Table:**

C_11_H_12_FNO	*F*(000) = 408
*M**_r_* = 193.22	*D*_x_ = 1.285 Mg m^−^^3^
Monoclinic, *P*2_1_/*c*	Melting point = 336–335 K
Hall symbol: -P 2ybc	Mo *K*α radiation, λ = 0.71073 Å
*a* = 13.2832 (6) Å	Cell parameters from 5766 reflections
*b* = 5.8530 (2) Å	θ = 3.5–29.0°
*c* = 14.2995 (8) Å	µ = 0.10 mm^−^^1^
β = 116.086 (6)°	*T* = 293 K
*V* = 998.49 (8) Å^3^	Block, yellow
*Z* = 4	0.3 × 0.2 × 0.2 mm

### Data collection

**Table:**

Oxford Diffraction Xcalibur Sapphire3 diffractometer	1952 independent reflections
Radiation source: fine-focus sealed tube	1430 reflections with *I* > 2σ(*I*)
Graphite monochromator	*R*_int_ = 0.040
Detector resolution: 16.1049 pixels mm^-1^	θ_max_ = 26.0°, θ_min_ = 3.5°
ω scan	*h* = −16→16
Absorption correction: multi-scan (*CrysAlis PRO*; Oxford Diffraction, 2010)	*k* = −7→7
*T*_min_ = 0.824, *T*_max_ = 1.000	*l* = −17→17
14183 measured reflections	

### Refinement

**Table:**

Refinement on *F*^2^	Primary atom site location: structure-invariant direct methods
Least-squares matrix: full	Secondary atom site location: difference Fourier map
*R*[*F*^2^ > 2σ(*F*^2^)] = 0.046	Hydrogen site location: inferred from neighbouring sites
*wR*(*F*^2^) = 0.133	H-atom parameters constrained
*S* = 1.04	*w* = 1/[σ^2^(*F*_o_^2^) + (0.0668*P*)^2^ + 0.1767*P*] where *P* = (*F*_o_^2^ + 2*F*_c_^2^)/3
1952 reflections	(Δ/σ)_max_ = 0.002
129 parameters	Δρ_max_ = 0.20 e Å^−^^3^
0 restraints	Δρ_min_ = −0.16 e Å^−^^3^

### Special details

**Table:**

Experimental. *CrysAlis PRO*, Oxford Diffraction Ltd., Version 1.171.34.40 (release 27–08-2010 CrysAlis171. NET) (compiled Aug 27 2010,11:50:40) Empirical absorption correction using spherical harmonics, implemented in SCALE3 ABSPACK scaling algorithm.
Geometry. All e.s.d.'s (except the e.s.d. in the dihedral angle between two l.s. planes) are estimated using the full covariance matrix. The cell e.s.d.'s are taken into account individually in the estimation of e.s.d.'s in distances, angles and torsion angles; correlations between e.s.d.'s in cell parameters are only used when they are defined by crystal symmetry. An approximate (isotropic) treatment of cell e.s.d.'s is used for estimating e.s.d.'s involving l.s. planes.
Refinement. Refinement of *F*^2^ against ALL reflections. The weighted *R*-factor *wR* and goodness of fit *S* are based on *F*^2^, conventional *R*-factors *R* are based on *F*, with *F* set to zero for negative *F*^2^. The threshold expression of *F*^2^ > σ(*F*^2^) is used only for calculating *R*-factors(gt) *etc*. and is not relevant to the choice of reflections for refinement. *R*-factors based on *F*^2^ are statistically about twice as large as those based on *F*, and *R*- factors based on ALL data will be even larger.

### Fractional atomic coordinates and isotropic or equivalent isotropic displacement parameters (Å^2^)

**Table:**

	*x*	*y*	*z*	*U*_iso_*/*U*_eq_	
C7	0.14077 (14)	0.3879 (3)	0.44568 (12)	0.0387 (4)	
F1	−0.19144 (9)	0.5459 (2)	0.27506 (10)	0.0802 (4)	
O1	0.28148 (11)	0.1122 (2)	0.52645 (12)	0.0752 (5)	
C1	0.26094 (15)	0.3183 (3)	0.50936 (13)	0.0463 (4)	
C2	0.34503 (14)	0.4916 (3)	0.54818 (13)	0.0457 (4)	
H2	0.3259	0.6447	0.5332	0.055*	
C3	0.45299 (14)	0.4299 (3)	0.60714 (13)	0.0489 (5)	
H3	0.4653	0.2738	0.6186	0.059*	
N4	0.54383 (12)	0.5600 (2)	0.65100 (12)	0.0548 (5)	
C5	0.53933 (17)	0.8047 (3)	0.63606 (18)	0.0656 (6)	
H5A	0.5800	0.8453	0.5972	0.098*	
H5B	0.5724	0.8793	0.7026	0.098*	
H5C	0.4627	0.8520	0.5985	0.098*	
C6	0.65389 (16)	0.4629 (4)	0.71403 (17)	0.0689 (6)	
H6A	0.6486	0.2993	0.7130	0.103*	
H6B	0.6810	0.5165	0.7844	0.103*	
H6C	0.7048	0.5088	0.6863	0.103*	
C8	0.05728 (15)	0.2340 (3)	0.43627 (13)	0.0460 (4)	
H8	0.0773	0.0921	0.4684	0.055*	
C9	−0.05434 (15)	0.2869 (3)	0.38041 (14)	0.0510 (5)	
H9	−0.1098	0.1845	0.3761	0.061*	
C10	−0.08165 (14)	0.4935 (3)	0.33139 (13)	0.0476 (5)	
C11	−0.00276 (15)	0.6494 (3)	0.33688 (13)	0.0468 (5)	
H11	−0.0240	0.7880	0.3018	0.056*	
C12	0.10921 (14)	0.5974 (3)	0.39551 (13)	0.0424 (4)	
H12	0.1638	0.7035	0.4014	0.051*	

### Atomic displacement parameters (Å^2^)

**Table:**

	*U*^11^	*U*^22^	*U*^33^	*U*^12^	*U*^13^	*U*^23^
C7	0.0408 (9)	0.0342 (8)	0.0392 (8)	−0.0007 (7)	0.0158 (7)	−0.0018 (7)
F1	0.0383 (7)	0.0777 (9)	0.1045 (10)	0.0076 (6)	0.0130 (7)	0.0085 (7)
O1	0.0530 (9)	0.0379 (7)	0.1041 (12)	0.0033 (6)	0.0066 (8)	0.0109 (7)
C1	0.0446 (10)	0.0361 (9)	0.0504 (10)	0.0021 (7)	0.0136 (8)	0.0031 (7)
C2	0.0405 (10)	0.0367 (9)	0.0522 (10)	0.0028 (7)	0.0134 (8)	0.0008 (7)
C3	0.0454 (11)	0.0380 (9)	0.0542 (10)	0.0017 (8)	0.0134 (8)	−0.0009 (8)
N4	0.0370 (8)	0.0433 (9)	0.0680 (10)	0.0025 (7)	0.0082 (7)	−0.0015 (7)
C5	0.0526 (12)	0.0458 (11)	0.0848 (15)	−0.0024 (9)	0.0179 (11)	−0.0022 (10)
C6	0.0416 (11)	0.0642 (13)	0.0804 (14)	0.0076 (10)	0.0080 (10)	0.0013 (11)
C8	0.0483 (10)	0.0364 (9)	0.0476 (10)	−0.0037 (7)	0.0159 (8)	0.0032 (7)
C9	0.0436 (10)	0.0489 (10)	0.0585 (11)	−0.0102 (8)	0.0206 (9)	−0.0012 (8)
C10	0.0363 (9)	0.0504 (10)	0.0501 (10)	0.0031 (8)	0.0134 (8)	−0.0041 (8)
C11	0.0483 (10)	0.0382 (9)	0.0496 (10)	0.0068 (8)	0.0175 (8)	0.0034 (7)
C12	0.0410 (9)	0.0368 (9)	0.0487 (9)	−0.0023 (7)	0.0191 (8)	0.0013 (7)

### Geometric parameters (Å, º)

**Table:**

C7—C8	1.389 (2)	C5—H5B	0.9600
C7—C12	1.389 (2)	C5—H5C	0.9600
C7—C1	1.505 (2)	C6—H6A	0.9600
F1—C10	1.355 (2)	C6—H6B	0.9600
O1—C1	1.237 (2)	C6—H6C	0.9600
C1—C2	1.428 (2)	C8—C9	1.375 (2)
C2—C3	1.354 (2)	C8—H8	0.9300
C2—H2	0.9300	C9—C10	1.365 (3)
C3—N4	1.328 (2)	C9—H9	0.9300
C3—H3	0.9300	C10—C11	1.365 (3)
N4—C5	1.445 (2)	C11—C12	1.382 (2)
N4—C6	1.454 (2)	C11—H11	0.9300
C5—H5A	0.9600	C12—H12	0.9300
			
C8—C7—C12	118.41 (16)	N4—C6—H6A	109.5
C8—C7—C1	118.20 (15)	N4—C6—H6B	109.5
C12—C7—C1	123.39 (16)	H6A—C6—H6B	109.5
O1—C1—C2	123.30 (16)	N4—C6—H6C	109.5
O1—C1—C7	117.81 (16)	H6A—C6—H6C	109.5
C2—C1—C7	118.89 (14)	H6B—C6—H6C	109.5
C3—C2—C1	119.05 (16)	C9—C8—C7	121.44 (16)
C3—C2—H2	120.5	C9—C8—H8	119.3
C1—C2—H2	120.5	C7—C8—H8	119.3
N4—C3—C2	129.38 (17)	C10—C9—C8	118.23 (17)
N4—C3—H3	115.3	C10—C9—H9	120.9
C2—C3—H3	115.3	C8—C9—H9	120.9
C3—N4—C5	121.90 (15)	F1—C10—C9	118.63 (16)
C3—N4—C6	121.72 (16)	F1—C10—C11	118.78 (16)
C5—N4—C6	116.35 (16)	C9—C10—C11	122.59 (16)
N4—C5—H5A	109.5	C10—C11—C12	118.80 (16)
N4—C5—H5B	109.5	C10—C11—H11	120.6
H5A—C5—H5B	109.5	C12—C11—H11	120.6
N4—C5—H5C	109.5	C11—C12—C7	120.49 (16)
H5A—C5—H5C	109.5	C11—C12—H12	119.8
H5B—C5—H5C	109.5	C7—C12—H12	119.8
			
C8—C7—C1—O1	19.2 (3)	C1—C7—C8—C9	179.18 (16)
C12—C7—C1—O1	−160.52 (17)	C7—C8—C9—C10	1.8 (3)
C8—C7—C1—C2	−159.98 (17)	C8—C9—C10—F1	179.16 (15)
C12—C7—C1—C2	20.4 (3)	C8—C9—C10—C11	−0.8 (3)
O1—C1—C2—C3	−0.3 (3)	F1—C10—C11—C12	179.12 (15)
C7—C1—C2—C3	178.80 (16)	C9—C10—C11—C12	−0.9 (3)
C1—C2—C3—N4	−179.93 (18)	C10—C11—C12—C7	1.6 (3)
C2—C3—N4—C5	−3.2 (3)	C8—C7—C12—C11	−0.6 (3)
C2—C3—N4—C6	178.8 (2)	C1—C7—C12—C11	179.03 (15)
C12—C7—C8—C9	−1.1 (3)		

### Hydrogen-bond geometry (Å, º)

**Table:**

*D*—H···*A*	*D*—H	H···*A*	*D*···*A*	*D*—H···*A*
C6—H6*B*···O1^i^	0.96	2.59	3.531 (3)	168

Symmetry code: (i) −*x*+1, *y*+1/2, −*z*+3/2.
